# Spondylocarpotarsal synostosis syndrome due to a novel loss of function *FLNB* variant: a case report

**DOI:** 10.1186/s12891-020-03890-2

**Published:** 2021-01-06

**Authors:** Samina Yasin, Outi Makitie, Sadaf Naz

**Affiliations:** 1 School of Biological Sciences, University of the Punjab, Quaid-i-Azam Campus, Lahore, Punjab Pakistan; 2 Children’s Hospital, University of Helsinki and Helsinki University Hospital, Helsinki, Finland; 3 Folkhälsan Institute of Genetics, Helsinki, Finland

**Keywords:** Carpal synostosis, Scoliosis, Exome sequencing, Pakistan, Case report

## Abstract

**Background:**

Loss of function or gain of function variants of Filamin B (*FLNB)* cause recessive or dominant skeletal disorders respectively. Spondylocarpotarsal synostosis syndrome (SCT) is a rare autosomal recessive disorder characterized by short stature, fused vertebrae and fusion of carpal and tarsal bones. We present a novel *FLNB* homozygous pathogenic variant and present a carrier of the variant with short height.

**Case presentation:**

We describe a family with five patients affected with skeletal malformations, short stature and vertebral deformities. Exome sequencing revealed a novel homozygous frameshift variant c.2911dupG p.(Ala971GlyfsTer122) in *FLNB*, segregating with the phenotype in the family. The variant was absent in public databases and 100 ethnically matched control chromosomes. One of the heterozygous carriers of the variant had short stature.

**Conclusion:**

Our report expands the genetic spectrum of *FLNB* pathogenic variants. It also indicates a need to assess the heights of other carriers of *FLNB* recessive variants to explore a possible role in idiopathic short stature.

**Supplementary Information:**

The online version contains supplementary material available at 10.1186/s12891-020-03890-2.

## Highlights

- We describe a novel homozygous variant in *FLNB*
- A biallelic *FLNB* variant causes SCT in five patients of a family
- Mild short stature of a carrier may indicate a semi-dominant less severe heterozygous effect of the variant

## Background

Spondylocarpotarsal synostosis syndrome (SCT; OMIM # 272460) is an autosomal recessive disorder. Patients with SCT are characterized by short stature, vertebral fusion, scoliosis or lordosis and synostosis of carpal and tarsal bones. Other skeletal deformities include clinodactyly, brachydactyly, limited joint mobility, cleft palate and dysmorphic facial features. Patients with SCT can exhibit dental enamel hypoplasia and mixed hearing loss [1, 2].

**Fig. 1:**
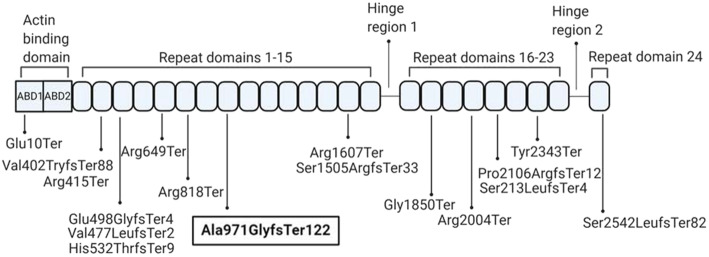
Schematic representation of the FLNB protein with all identified homozygous variants causing recessive spondylocarpotarsal synostosis syndrome. Variant identified in the present study is shown in bold. ABD1 and ABD2 -Actin binding domains. Twenty-four repeat domains are separated by two hinge regions; hinge 1 and hinge 2

Filamins arrange actin into three dimensional networks and control structure and function of cytoskeleton. Filamin B, encoded by *FLNB* is involved in cartilage growth and condensation of developing vertebrae [2]. FLNB is composed of two actin-binding domains (ABD) at the N-terminus and 24 filamin repeat regions. There are two hinges interrupting these repeat regions (Fig. 1) [3].

SCT can be inherited due to *MYH3* variant*,* or predominantly by biallelic loss of function alleles of *FLNB* [1, 4]. Interestingly, heterozygous missense variants of *FLNB* cause different autosomal dominant syndromes which include Boomerang dysplasia (BD; OMIM # 112310), Larsen syndrome (LS; OMIM # 150250), Atelosteogenesis I (AOI; OMIM # 108720) and III (AOIII; OMIM # 108721) [2]. Less than 20 families having patients with *FLNB*-related SCT are known [3]. We report a large family with SCT due to a novel homozygous frameshift variant of *FLNB*.

## Case presentation

### Subjects and methods

**Fig. 2:**
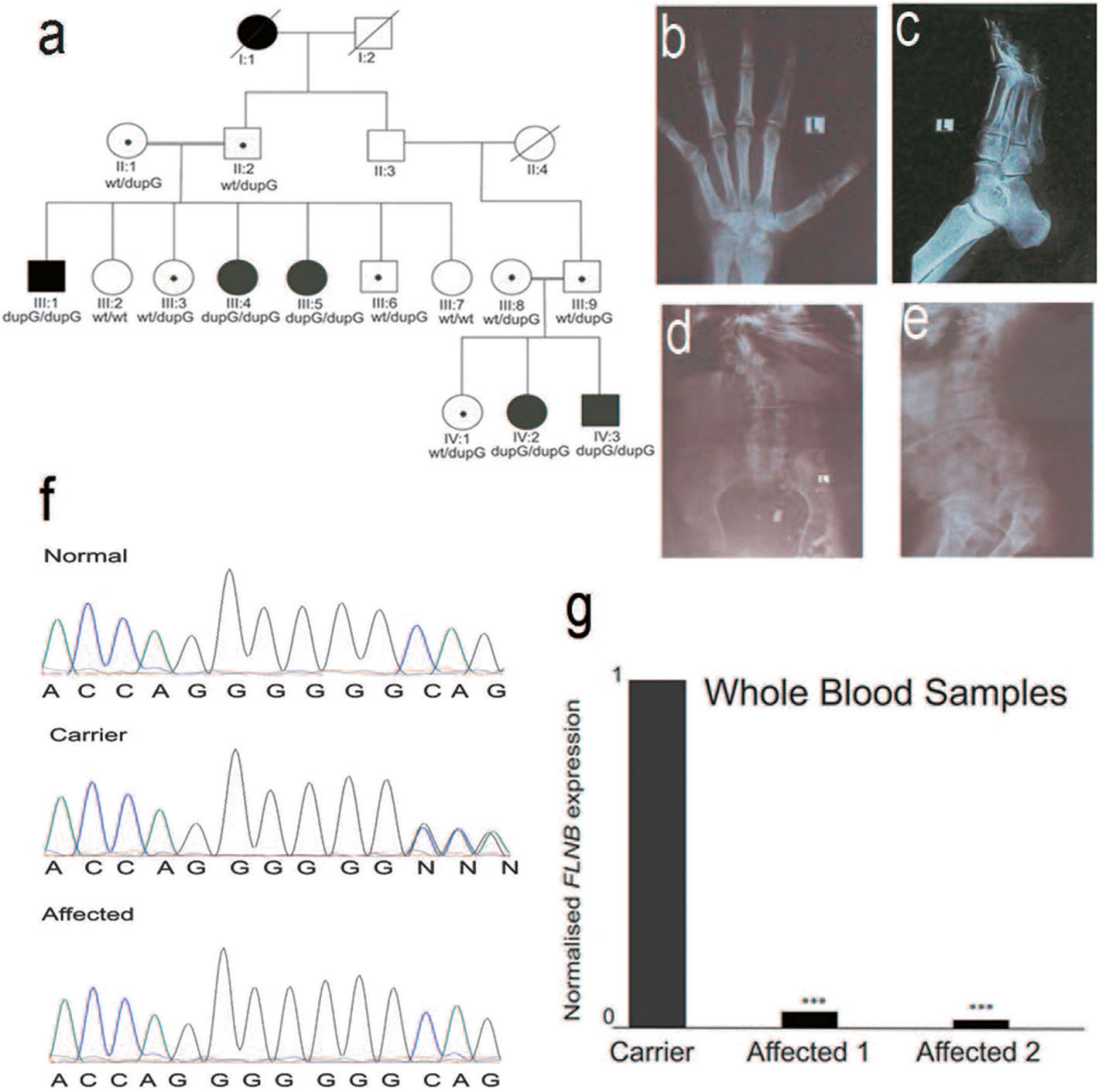
Pedigree of family SYD07 and radiographs of individual III:1 at 32 years of age. **a** Pedigree: Black symbols indicate affected individuals. Genotypes of the identified variant for all participants are provided below the symbols. Carriers of the variants are depicted with black dots inside the circles or squares. **b** Radiographs of patient III:1: X-Ray of left hand showing capitate hamate (Carpal bones) fusion. **c** Anteroposterior view of the left foot showing tarsal fusion. **d** Anteroposterior view of the spine showing scoliosis with fusion of vertebrae at thoracic and lumbar region. **e** Lateral view of the spine showing lordosis. **f** Chromatogram of the *FLNB* showing c.2911dupG variant. Arrows indicate the point of variant from wild type to heterozygous carrier and in homozygous affected individual. **g** RT qPCR from whole blood samples of an unaffected heterozygous carrier and two homozygous affected individuals in the family SYD07 demonstrates that the variant c.2911dupG decreases *FLNB* expression. Significant fold change (*p* < 0.05, *p* = 0.0003) is denoted with three asterisks

A family SYD07 (Fig. 2a) with two consanguineous couples having five individuals affected with short stature and skeletal malformations was recruited from Punjab. Blood samples were collected from all participants. Genomic DNA was extracted from whole blood according to a standard protocol involving sucrose lysis and salting out.

Exome sequencing was performed on the DNA sample of a severely affected patient III:1 at 100X with SureSelect V5-post capture on Illumina platform sequencer (Macrogen, South Korea). WES data was annotated using wANNOVAR (http://wannovar.usc.edu/index.php). Variants were sorted against 1000 g2015, ESP6500 ExAc03 and gnomAD data. Variants with an allele frequency of lower than 0.01 were retained. Homozygous exonic and splice site variants were selected. Candidate variant selected after analysis of exome sequencing data was confirmed by Sanger sequencing of *FLNB* (NM_001164317.1) exon 20.

Cell lines from the members of family with SCT were not available. Therefore, we obtained blood samples of two affected individuals and one unaffected carrier from family SYD07. RNA was extracted using TRIzol reagent (Thermo Fisher Scientific). Random primed cDNA libraries were synthesized using RevertAid First strand cDNA synthesis kit (Thermo Fisher Scientific). For qualitative PCR, two sets of primers were designed for amplification of partial *FLNB* cDNA fragments; An Outer 5ˊ- CATCTCAGGAAATCCCCGCCAGCC, 5ˊ -GGTAAGAGACGGAGCAGGTCCCATC pair and an Inner 5ˊ -CACCCACCCAACAGGGCAACATGC, 5ˊ- GATGGTGAACTCGGCAGGCTTGCC pair. After PCR with the outer set of primers on cDNA libraries, a nested PCR was performed on the diluted product of 822 bp obtained from the first reaction, using the inner set of primers for a final 458 bp product. *GAPDH* primer pair of RevertAid First strand cDNA synthesis kit (Thermo Fisher Scientific) was used as a positive control.

For quantitative real time PCR (RT qPCR), one *FLNB* specific 5ˊ- AATGGGCTGGAAAACAGGGTG, 5ˊ- CAGGTGTCACTAGGCATGGC primer pair and one housekeeping gene *GAPDH* specific 5ˊ- CGTGGAAGGACTCATGACCA, 5ˊ- GGATGACCTTGCCCACAGC primer pair were designed. Power SYBR Green Master Mix (Thermo Fisher Scientific) was used for amplification of respective cDNA fragments from an equal input of cDNA libraries of all family members in triplicate on cycler PikoReal 96 (Thermo Fisher Scientific). Each experiment was repeated thrice independently.

### Statistical analysis

Triplicate quantification cycle (Cq) values also called threshold-cycle (Ct) values were averaged and normalised to the control *GAPDH* values for each sample. Normalised expression of *FLNB* in the unaffected carrier individual was set to 1. ΔCq and ΔΔCq values were calculated to determine the fold change of *FLNB* in the affected patients. Statistical analysis was done by one-way analysis of variance (ANOVA) Tukey’s Multiple Comparison Test to calculate significant fold change (*p* values).

### Clinical findings

The five affected individuals ranged in age from 7 to 32 years and their heights were 132 cm (5.1 SD), 125 cm (− 5.0 SD), 128 cm (− 4.6 SD), 119 cm (− 4.3 SD) and 108 cm (− 3.4 SD) (Table 1). Heights of the parents (II:2, III:8, III:9) were near or above average (167 cm,152 cm and 179 cm with SDs ranging from − 1 to + 1) except a mother (II:1) who had a height of 147 cm (− 1.8 SD).

**Table 1:** Clinical manifestations in affected individual of family SYD07

Abnormalities	Patients				
	III:1	III:4	III:5	IV:2	IV:3
**Age (Years)**	32	26	24	11	7
**Sex**	Male	Female	Female	Female	Male
**Height (cm) with**	132	125	128	119.	108
**Standard Deviation (SD)**	−5.1	−5.0	−4.6	−4.3	−3.7
**Short Stature**	+	+	+	+	+
**Short trunk**	+	+	+	+	+
**Short neck**	+	+	+	+	+
**Hearing deficit**	+	+	–	–	–
**Vision impairment**	–	–	+	–	–
**Dysmorphic face**	+	–	–	+	+
**Flat feet**	–	–	–	–	+
**Joint mobility limitation**	+	+	–	–	–

(+ present, − absent)

Radiographs of patient III:1 revealed fusion of capitate-hamate bones in the hand and tarsal fusion in the feet (Fig. 2b, c). Vertebral radiographs revealed scoliosis, lordosis and disrupted vertebral segmentation (Fig. 2d, e). Neck and trunk were short due to fusion of the vertebrae at cervical and thorax regions (Fig. 2d). The age of onset of visible skeletal malformations was reported to be 7 to 10 years in all affected participants. The early features included short stature and protruding chest. In addition, they had dysmorphic facial features with mild frontal bossing, anteverted nostrils and low set ears, short necks, and spinal deformities (Table 1). Progressive hearing loss, vision impairment, flat feet and limited joint movement were also observed in some of the patients (Table 1).

### DNA analysis and RNA expression

Analysis of exome sequencing data for individual III:1 revealed 34 homozygous variants after applying the filtration criteria (Table S1). We next discarded variants for which a number of individuals were homozygous in the public databases, the affected amino acids were not conserved in different vertebral orthologues or the variants were predicted to be benign by multiple software. This further reduced the number of variants to eight. Among these, the *FLNB* variant c.2911dupG p.(Ala971GlyfsTer122) (NM_001457.3) segregated with the disease phenotype and was homozygous in all affected members and heterozygous in the obligate carriers (Fig. 2f). This variant was absent from 100 ethnically matched control chromosomes as well as from public databases such as gnomAD (https://gnomad.broadinstitute.org/gene/ENSG00000136068?dataset=gnomad_r2_1), 1000 genomes (https://www.internationalgenome.org/data-portal/search?q=Ala971GlyfsTer122) GME (http://igm.ucsd.edu/gme/data-browser.php) and HGMD (http://www.hgmd.cf.ac.uk/ac/all.php). The variant has been submitted to the LOVD database (https://databases.lovd.nl/shared/variants/0000600855).

The c.2911dupG variant located within exon 20 creates a frameshift and introduces a premature stop codon, affecting domain 8 of FLNB, and if translated, predicts a 1511 residue shortened protein. Qualitative analysis using nested PCR demonstrated that *FLNB* specific products could be obtained from cDNA libraries prepared from blood samples of both a heterozygous carrier and the homozygous affected individuals (data not shown). However, quantitative real time PCR revealed that the amount of *FLNB* specific RNA was significantly reduced (*p* < 0.05, *p* = 0.0003) in both affected individuals as compared to the levels in the unaffected carrier (Fig. 2g).

## Discussion and conclusion

SCT syndrome is a rare disorder involving fusion of vertebrae accompanied by fusion of carpal and tarsal bones. SCT was first recognized in 1973 by Jones et al. with carpal synostosis and fused vertebrae. Other clinical reports revealed autosomal recessive cases comprising of scoliosis along with carpel fusion and block vertebrae [5, 6]. This condition was named as spondylocarpotarsal synostosis syndrome [7] and the causative gene for the syndrome was localized to chromosome 3p14 [8]. Biallelic variants in *FLNB* underlying this condition were reported in the same year [9]. SCT was described to be caused by truncation of FLNB in its repeat domains or loss of function of the protein. Additional missense, nonsense and frameshift variants were reported subsequently (Table 2) [1, 3, 10, 11].

**Table 2:** Comparison of clinical features of all patients having biallelic *FLNB* variants

Study	Variant	Gender (Ma:F)	Short stature	Short neck	Carpal fusion	Scoliosis	Facial deformities	Tarsal fusion	Hearing deficit	Lordosis	Enamel hypoplasia
(Krakow et al., 2004) [9]	c.1945C > T c.2452C > T c.4819C > T c.6408delC c.7029 T > G	5:6	11/11	9/11	11/11	11/11	11/11	7/11	7/11	NR	7/11
(Farrington-Rock et al., 2007) [10]	c.4671G > A c.5548G > T	2:0	2/2	2/2	NR	2/2	NR	NR	NR	NR	NR
(Mitter et al., 2008) [11]	c.6010C > T	1:0	1/1	1/1	1/1	1/1	1/1	NR	1/1	0/1	NR
(Yang et al., 2017) [1]	c.7621dupG	0:2	2/2	2/2	2/2	2/2	0/2	0/2	1/2	2/2	0/2
(Salian et al., 2018) [3]	c.28G > T c.429delinsCT c.1204delG c.1243C > T c.1493delA c.1592dup c.6317delC	6:4	10/10	6/10	9/10	9/10	3/10	3/10	2/10	2/10	NR
**Present study**	c.2911dupG	3:2	5/5	5/5	4/5	3/5	3/5	3/5	1/5	3/5	0/5
**Summary of features**	All nonsense or frameshift variants	17:14	100%	93%	90%	90%	54%	41%	38%	22%	22%

*NR* Not reported

FLNB is comprised of 24 repeat domains. Autosomal dominant disorders (BD/LS/AOI/AOIII) of *FLNB* are caused by heterozygous missense variants, which are mostly clustered in actin-binding domains and repeat domains 13–15. They result due to gain-of-function of FLNB. Recently, co-inheritance of some *FLNB* variants with variants in other genes important in skeletogenesis, were also identified to have a role in the complex trait of adolescent idiopathic scoliosis [12]. However, the recessively inherited SCT is caused by biallelic missense, nonsense or frameshift truncating variants, which result in loss of function of FLNB [1, 13]. Previous studies have shown that SCT is caused generally by variants affecting FLNB actin-binding domains and repeat domains [3] and specifically repeat regions 2, 3, 5, 6, 13, 14, 17, 19, 20, 22, 23 and 24. The novel frameshift variant identified in this study is located within the exon 20 encoding repeat region 8 of FLNB. Frameshift *FLNB* variants usually result in the absence of the encoded protein, mostly due to nonsense-mediated decay of mRNA [1]. Our results on blood samples also show that the frameshift variant will mark the mRNA for nonsense mediated decay. To our knowledge, this is the first report in which effect of a frameshift variant on *FLNB* RNA has been determined on a sample directly obtained from SCT patients.

To date 31 genetically characterized patients affected with autosomal recessive SCT from 15 families (including those presented here) harboring 17 biallelic variants of *FLNB* have been reported (Fig. 1). Clinical symptoms of up to 31 SCT patients have been described in detail [1, 11]. A short stature, short neck, scoliosis and carpal fusion are exhibited by most of the patients while other disease manifestations are more variable (Table 2). In the current study, the patients exhibited many of the clinical features associated with SCT. They had short statures with short necks and trunks, mild facial dysmorphism and progressive hearing loss. These manifestations were accompanied by scoliosis, lordosis, fusion of vertebrae and synostosis of carpal and tarsal bones. Capitate hamate synostosis was also present.

The less frequent characteristics of SCT include brachydactyly, clinodactyly, club feet, cleft palate and enamel hypoplasia. These conditions have been reported in less than 25% reported cases [1, 6, 14]. Rarely, rib anomalies, odontoid aplasia and epiphyseal dysplasia with SCT are also present [1, 2, 15]. None of these conditions were manifested by our patients.

In this study, the obligate carriers had the expected heights for their age and sex. However, one female (II:1) who was heterozygous for the variant exhibited mild short stature (147 cm, − 1.8 SD) without other symptoms, indicating a possible association of some *FLNB* heterozygous variants with milder growth impairment. Previously, heterozygous effect for a loss of function allele has been reported for one obligate carrier who had short stature and unilateral hip dysplasia [11]. It was hypothesized that this could be a nonspecific finding and further research was required. Our work also suggests the need for careful evaluation of carriers of recessively inherited *FLNB* alleles in order to categorically prove or disprove an incompletely penetrant heterozygous effect of the loss of function variants. Another hypothesis is that some carriers of *FLNB* variants may have additional variants in other gene/s important for skeletogenesis and an oligogenic mode of inheritance may be responsible for their short stature.

In conclusion, we present a family with autosomal recessive SCT and report a novel frameshift variant which is predicted to result in complete loss of function of *FLNB* due to nonsense mediated decay of the mRNA. The family has one heterozygous carrier with mild short stature which may indicate a semi-dominant less severe heterozygous effect of the variant or co-inheritance of pathogenic variants in other genes. Our report broadens the genetic spectrum of pathogenic alleles of *FLNB*.

## Supplementary Information

**Additional file 1: Table S1.** Variants revealed after whole exome sequence analysis and filtration.

## Data Availability

The variant number at LOVD data base is 0000600855 (https://databases.lovd.nl/shared/variants/0000600855). Supplementary data listing filtered variants of exome data is available as Table S1.

## Abbreviations

SCT: Spondylocarpotarsal synostosis syndrome
ABD: Actin-binding domain
FLNB: Filamin B
SD: Standard deviation
ANOVA: Analysis of variance
RT qPCR: Quantitative Real Time PCR
